# Improvements in hand function after intensive bimanual training are not associated with corticospinal tract dysgenesis in children with unilateral cerebral palsy

**DOI:** 10.1007/s00221-014-3889-x

**Published:** 2014-03-13

**Authors:** Kathleen M. Friel, Hsing-Ching Kuo, Jason B. Carmel, Stefan B. Rowny, Andrew M. Gordon

**Affiliations:** 1 Burke-Cornell Medical Research Institute, 785 Mamaroneck Avenue, White Plains, NY 10605 USA; 2 Department of Neurology, Brain Mind Research Institute, Weill Cornell Medical College, New York, NY 10021 USA; 3 Department of Biobehavioral Sciences, Teachers College of Columbia University, New York, NY 10027 USA; 4 Division of Experimental Therapeutics, Department of Psychiatry, Columbia University Medical Center, New York, NY 10032 USA; 5 Department of Pediatrics, Weill Cornell Medical College, New York, NY 10021 USA; 6 Department of Rehabilitation Medicine, Columbia University Medical Center, New York, NY 10032 USA

**Keywords:** Bimanual training, Rehabilitation, Corticospinal, Motor development

## Abstract

Unilateral cerebral palsy (CP) results from damage to the developing brain that occurs within the first 2 years of life. Previous studies found associations between asymmetry in the size of the corticospinal tract (CST) from the two hemispheres and severity of hand impairments in children with unilateral CP. The extent to which CST damage affects the capacity for hand function improvement is unknown. This study examines the association between an estimate of CST dysgenesis and (1) hand function and (2) the efficacy of intensive bimanual training in improving hand function. Children with unilateral CP, age 3.6–14.9 years, *n* = 35, received intensive bimanual training. Children engaged in bimanual functional/play activities (6 h/day, 15 days). Peduncle asymmetry, an estimate of CST dysgenesis, was measured on T1-weighted magnetic resonance imaging scans. Hand function was measured pre- and post-treatment using the assisting hand assessment (AHA) and Jebsen–Taylor test of hand function (JTTHF). AHA and JTTHF improved post-treatment (*p* < 0.001). Peduncle asymmetry was correlated with baseline AHA and JTTHF (*p* < 0.001) but not with AHA or JTTHF improvement post-training (*R* ^2^ < 0.1, *p* > 0.2). An estimate of CST dysgenesis is correlated with baseline hand function but is a poor predictor of training efficacy, possibly indicating a flexibility of developing motor systems to mediate recovery.

## Introduction

Poor function in the affected hand is among the greatest functional impairments for children with unilateral cerebral palsy (CP) (Gordon and Friel 2009). Whereas multiple neural systems help control hand function, the corticospinal system predominates for skilled voluntary movement in humans. Previous studies have demonstrated a strong association between asymmetry in the size of the corticospinal tract (CST) or cerebral peduncles and various features of hand function in children with unilateral CP (Bleyenheuft et al. 2007; Duque et al. 2003) and adult stroke patients (Barnes et al. 2008; Pineiro et al. 2000). Peduncle asymmetry is an estimate of CST dysgenesis, since the CST passes through the peduncles. Peduncle asymmetry is a consequence of the extent of initial damage to the motor system during development.

Asymmetry in the size of the CST, measured using diffusion tensor imaging (DTI) (Bleyenheuft et al. 2007; Yoshida et al. 2010) or by cross-sectional area of the cerebral peduncles (Bouza et al. 1994; Duque et al. 2003), is strongly correlated with stereognosis, and a measure of hand function in activities of daily living in children with unilateral CP. The timing of grip forces during a lifting task was also associated with peduncle asymmetry in children with unilateral CP (Duque et al. 2003). Another study found a correlation between peduncle asymmetry and stereognosis in children with unilateral CP (Bleyenheuft et al. 2007).

Intensive bimanual training has shown efficacy in improving hand function in children with unilateral CP (Facchin et al. 2011; Gordon et al. 2007, 2008, 2011; Sakzewski 2012; Sakzewski et al. 2011). However, the neuroanatomical substrate required for this improvement has not been examined. These therapies require great time and effort and are resource-consuming for patients, families, and clinicians. It is important to identify predictors of efficacy, so that therapy can be targeted to children who are most likely to benefit from it.

Two recent studies have examined associations between magnetic resonance imaging (MRI) features and efficacy of constraint-induced movement therapy (CIMT) in children with unilateral CP and adults with chronic stroke. Rocca et al. (2013) reported that fiber integrity of the lesion area was predictive of improvement in quality of upper extremity movement (Rocca et al. 2013). In a similar study, associations were found between baseline arm function and integrity of the CST on the affected side, as measured by DTI (Rickards et al. 2013). However, integrity of the CST was not predictive of improvement in arm function after CIMT in children or adults with unilateral CP or stroke.

There were two goals to this study. We first aimed to examine the association between peduncle asymmetry and bimanual and unimanual hand function. We next sought to examine the relationship between peduncle asymmetry and efficacy of bimanual training for improving hand function. We hypothesized that peduncle asymmetry would be associated with amount of improvement in hand function shown by children after bimanual therapy—those with large asymmetry were predicted to have less functional recovery. If an estimate of dysgenesis of the CST is associated with efficacy of therapy, MRI could inform decisions about which treatment is most appropriate for individual children with unilateral CP (Z’Graggen et al. 1998; Carmel et al. 2013).

## Methods

### Participants

Thirty-five children participated in this study. All participants had been diagnosed with congenital unilateral CP before the age of 1 year. Demographics of study participants are summarized in Table 1. Participants were included in the bimanual training protocol in accordance with previously established criteria (Gordon et al. 2011). Briefly, participants were required to have the ability to extend the wrist at least 20° and the fingers at the metacarpophalangeal joint at least 10° from full flexion in the more-affected hand. Participants were also required to have the ability to lift the more-affected arm 15 cm from a table and to grasp objects, to have a greater than 50 % asymmetry in Jebsen–Taylor test of hand function (JTTHF) scores between the two hands, and be mainstreamed in school with a Kaufman brief intelligence score >70. Potential participants with contraindications to MRI (e.g., metallic implants) were also excluded.

**Table 1:** Demographic and clinical measures (±SD)

Measure	
*n*	35
Age	7.9 ± 2.9
Gender	12 F, 23 M
Pre-training JTTHF (s)	294.2 ± 220.0
Post-training JTTHF (s)	199.3 ± 163.2
% Improvement JTTHF	31.1 ± 21.8
Pre-training AHA (AHA unit)	61.0 ± 11.3
Post-training AHA (AHA unit)	64.0 ± 12.1
% Improvement AHA	58.4 ± 62.3
Side of lesion	15 R, 20 L
Peduncle asymmetry	73.8 ± 14.6
Lesion type	6 CM
	20 PV
	9 C/SC

*CM* cortical malformation, *PV* periventricular injury, *C/SC* cortical/subcortical lesion

Participants were recruited for the bimanual training study from clinics in the NYC area, our center website (http://www.tc.edu/centers/cit/), ClinicalTrials.gov (NCT00305006), and online parent forums. All research was approved by the Institutional Review Boards of Teachers College and Columbia University Medical Center. Informed assent/consent was obtained from all participants and their parents. Some participants (18 out of 35) were recruited for a randomized clinical trial testing the efficacy CIMT and HABIT, and their motor outcomes have been published (Gordon et al. 2011). In these participants, we received permission to acquire an MRI or analyze a previously obtained MRI after the children had participated in the intervention. An additional 17 participants were recruited after the published RCT had been completed and received HABIT training as described below (Gordon et al. 2011).

### MRI acquisition and measurement

Magnetic resonance imagings were obtained in one of two ways. For 10 participants, parents were provided a copy of an MRI that the child had received as part of their clinical care. For these children, the average age when the MRI was performed was 24.7 ± 27.6 months (range 0–90 months, median 13.8 months). Eight of the ten children were 2 years of age or younger at the time of the MRI). T1-weighted scans were used in this study.

For an additional 25 participants, T1-weighted MRI scans were obtained at the Program for Imaging and Cognitive Sciences facility at Columbia University Medical Center (CUMC), using a 3T Phillips magnetic resonance scanner with a six-channel head coil. Before the MRI was performed, a physician administered a safety screening evaluation. Images with a 2 mm^3^ voxel size were acquired in the axial plane with scan duration of 297 s. For these children, the average age at the time of the scan was 9.1 ± 3.0 years.

The size of the cerebral peduncles was measured at the level of the rostral midbrain, as indicated by the presence of the mammillary bodies. Analysis was performed on a Macintosh computer using the public domain NIH image program (http://rsb.info.nih.gov/nih-image/). NIH image is a versatile program that has been shown to be accurate and efficient in analyzing structural features of MRI scans^19^. Since it was not possible to dissociate the substantia nigra from the peduncles, the substantia nigra was included in the measure, which encompassed the area from the lateral sulcus to the interpeduncular fossa. The borders of the outlined area could be finely adjusted in the software program to maximize accuracy of tracing. The software provided an areal measure of the outlined territory, which was converted to mm^2^ using scale bars on the image. An example of the measurement of the peduncle (outlined) is shown in the inset of Fig. 2. Asymmetry in peduncle size was calculated as a ratio of the area of the more-affected peduncle divided by the area of the less-affected peduncle × 100 %. A lower number indicates a greater difference in the sizes of the peduncles.

For part of our analysis, we used a normalized peduncle asymmetry measure. We normalized peduncle size to the circumference of the brain in an axial slice, taken at the level of largest brain circumference. An MRI viewing program (OsiriX, Pixmeo Sarl) was used to scroll through each child’s MRI from dorsal to ventral. At the level of maximum brain circumference, the image was saved and exported to NIH image. Brain circumference was measured by tracing the outline of the brain on the image. The software provided the area of the outlined brain cross section. If it was visually unclear which section contained the brain at maximum circumference, several sections were exported and measured in NIH image. The largest circumference from the series was used in the calculation of normalized peduncle asymmetry. The raw peduncle asymmetry was divided by the cross-sectional area of the brain at the point of maximum circumference.

Lesion type was determined for all cases. For statistical comparisons, lesion type was categorized as cortical malformation (CM), periventricular injury (PV), or cortical/subcortical lesion (C/SC) and is described in detail in Table 2. For participants who received MRIs at CUMC, a pediatric neurologist categorized the lesion type. For MRIs that were obtained as part of the child’s clinical care, lesion type was obtained from the MRI report and verified by a pediatric neurologist. For subjects who had more than one scan, the most recent scan was used for the measurements and analysis.

**Table 2:** Peduncle asymmetry remains stable over time

Subject	Age at MRI (years months)	Peduncle asymmetry	Percent difference
1	1.11	1.10	−3.7
	6.11	1.06	
2	1.11	1.38	−8.3
	7.11	1.27	
3	0.6	1.34	+3.6
	0.9	1.39	
4	0.9	1.44	−7.1
	1.2	1.43	
	1.10	1.34	

### Intensive bimanual training

Intensive bimanual training [hand–arm intensive bimanual training (HABIT)] was performed to improve hand and arm function. HABIT employs principles of motor learning, including repetitive practice and skill progression to engage the more-affected hand in dexterous activities. In HABIT, participants were actively engaged in bimanual tasks during the training period. HABIT was conducted in a day-camp setting using child-friendly activities. Seven HABIT camps were performed from July 2007 to July 2012. Every participant received 90 h of treatment over 3 weeks (6 h/day, 15 days). HABIT was conducted one-to-one with a trained interventionist and supervised by a physical or occupational therapist. Details of HABIT procedures are presented elsewhere (Gordon et al. 2011).

### Assisting hand assessment (AHA)

The AHA is a standardized test that quantifies the effectiveness of assisting hand use in performing bimanual activities in children with unilateral upper limb disabilities. The AHA has excellent validity and reliability (0.97–0.99) and responsiveness to change. The test was videotaped and scored off-site by an experienced blinded evaluator. Transformed logit data (AHA unit) were analyzed.

### Jebsen–Taylor test of hand function (JTTHF)

Jebsen–Taylor test of hand function for the affected hand was the primary outcome measure (Jebsen et al. 1969). JTTHF is a standardized test that quantifies the time to complete skilled tasks. It consists of functional subtests including card flipping, small objects manipulation and placement, simulated eating, checker stacking, and empty and full can manipulation. Nonetheless, the JTTHF is considered a measure of hand dexterity, as movement speed is a correlate of how well a child can use the hand. Each child was evaluated prior to and within 2 days of completing treatment.

### Statistical analyses

A paired *t* test was used to evaluate changes in JTTHF and AHA before and after treatment using SPSS (IBM, version 21). Associations between peduncle asymmetry and JTTHF measures were evaluated using linear regression analyses. Regression models were generated in SPSS. First, we generated models between one dependent and one independent variable. Then, the following covariates were added to regression models as noted in the results: initial severity, age, gender, lesion type, side most affected by the lesion, and the lag time between when the MRI was taken and when the child participated in the hand training. These covariates were chosen because it was suspected that these variables could affect the relationship between the dependent and independent variables. Correlation coefficients and *R* ^2^ values are reported. *p* values less than 0.05 were considered statistically significant.

## Results

### Study participants

Table 1 summarizes the demographic and clinical features of participants. Lesion type was categorized as periventricular, cortical/subcortical, and CM. Of the children with cortical and subcortical damage, three had a lesion restricted to the sensorimotor cortex. The other children all had involvement of the basal ganglia and parietal lobe. Although all children had a diagnosis of unilateral CP, bilateral damage was found on the MRI of five children. Due to the heterogeneity of lesion type among participants in the study, we are unable to draw conclusions on the impact of lesion location on motor function and capacity for recovery.

For some children, the MRI was done several years before the child participated in the hand training protocol. Lag time between MRI time and training (average 42.5 months, SD = 33.0, range 0–111.7 months) was added to the regression models that are presented below.

### Bimanual training improves hand function

**Fig. 1:**
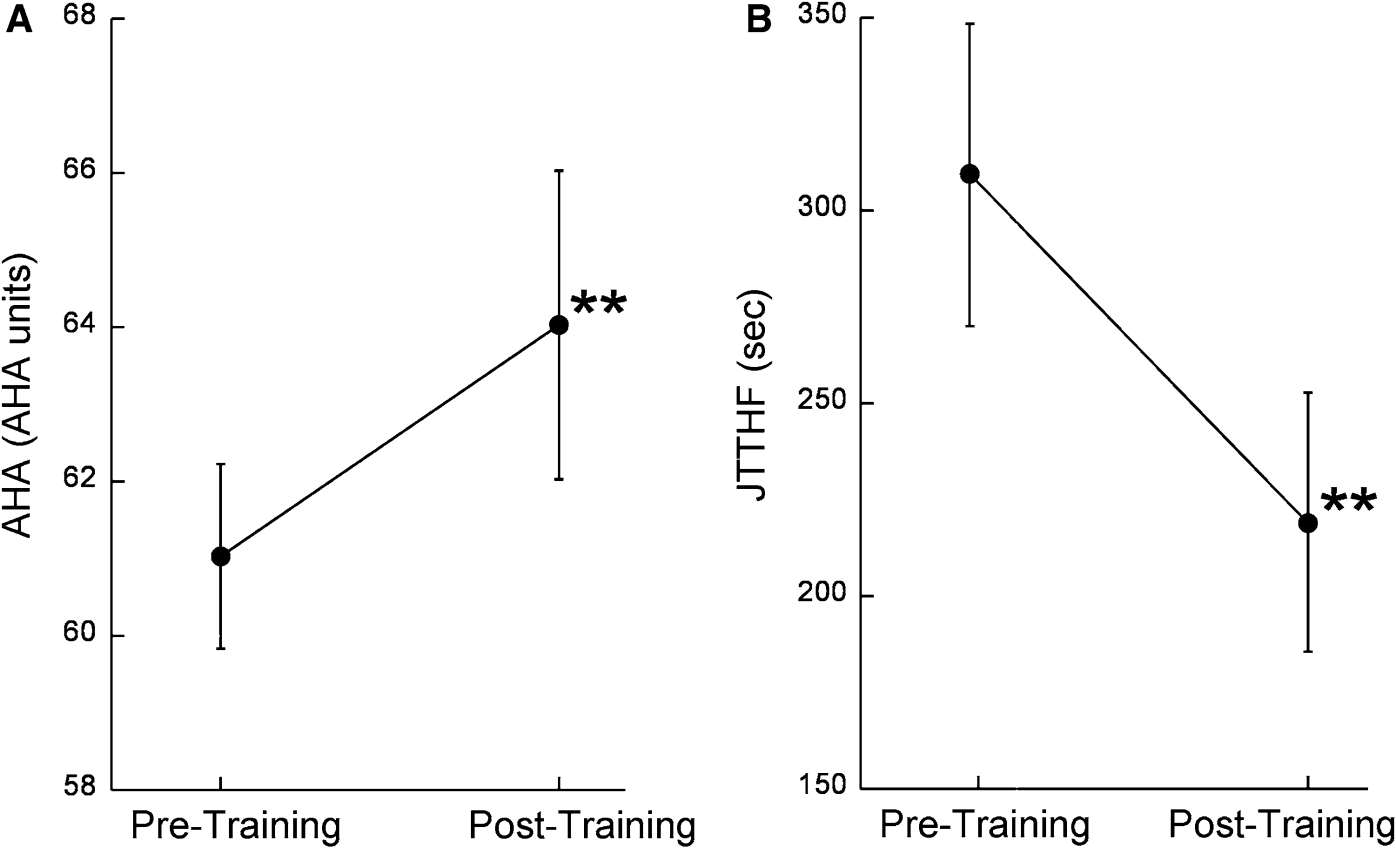
Intensive bimanual therapy improves **a** bimanual and **b** unimanual hand function (*p < 0.001). *Error bars* represent SEM

Some participants (*n* = 18) in this study were included in a randomized clinical trial that demonstrated efficacy of HABIT in improving unimanual and bimanual hand use, and their motor outcomes have been published (Gordon et al. 2011). Figure 1 shows change in bimanual use (AHA) and unimanual capacity (JTTHF) after HABIT in all participants of the current study. There was a significant improvement in AHA score (Fig. 1a, *df* = 33, *t* = 4.41, *p* < 0.0001) and JTTHF score (Fig. 1b, *df* = 33, *t* = 4.47, *p* < 0.0001) after HABIT.

### Stability in peduncle asymmetry measures between raters and across time

Peduncle measurements were performed by analysts (KF, HK) blinded to behavioral data at the time of performing analysis. Inter-rater reliability was tested by comparing measures done independently by two different analysts, both blinded to behavioral data, on a subset of the MRIs (*n* = 15). Pearson’s correlation between the measures of the two raters was 0.95 (95 % CI 0.85–0.98). Among these images measured by two analysts were five repeated MRI scans done on two different children at different ages. The Pearson’s correlation between measures of the two analysts for the repeated MRIs was 0.96 (95 % CI 0.84–0.99).

Four children had MRI scans at more than one time point. Table 2 summarizes peduncle asymmetry measured from each of the scans. In all four children, the first MRI scan was performed when the child was less than 2 years of age. The percent difference in peduncle asymmetry measured from the different scans was 3.6–8.3 %. Although repeat scans were obtained on only a small number of children, these findings indicate that peduncle asymmetry shows low variability between scans, even when scan times are separated by many years. The stability in asymmetry suggests that there is little change in the CST between these developmental time points.

### Peduncle asymmetry is associated with baseline hand function

**Fig. 2:**
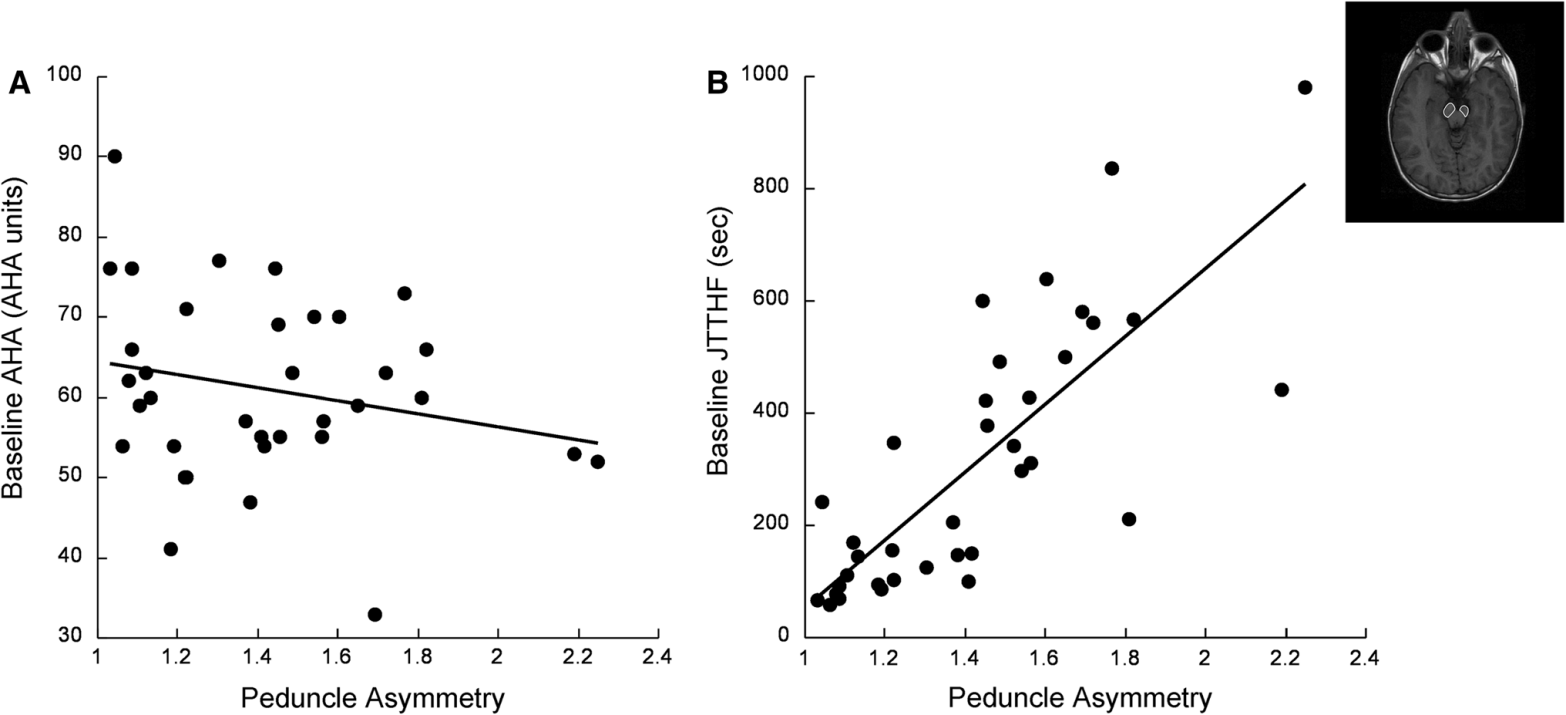
Peduncle asymmetry predicts baseline **a** bimanual and **b** unimanual hand function. Pre-treatment AHA and peduncle asymmetry were positively linearly associated [*F*(1,33) = 12.56.93, *p* = 0.01, *R* ^2^ = 0.28]. Pre-treatment JTTHF and peduncle asymmetry were strongly and positively linearly associated [*F*(1,33) = 50.93, *p* < 0.0001, *R* ^2^ = 0.6]. *Inset* T1-weighted MRI showing cerebral peduncles, outlined

We examined the linear association between baseline hand function and peduncle asymmetry. There was a significant linear correlation between baseline AHA score and peduncle asymmetry [Fig. 2a, *F*(1,33) = 12.56, *p* = 0.01, *r* = 0.53, *R* ^2^ = 0.28]. Greater asymmetry was associated with poorer bimanual hand use before the intervention. There was a significant linear correlation between baseline JTTHF and peduncle asymmetry [Fig. 2b, *F*(1,33) = 50.33, *p* < 0.0001, *r* = 0.78, *R* ^2^ = 0.60]; greater asymmetry was associated with poorer unimanual capacity before the intervention.

We examined the contribution of the following covariates to the linear relationship between peduncle asymmetry and baseline AHA and JTTHF: age, gender, side of lesion, lesion type, and the lag time from when the MRI was done to the time of training. None of these covariates were independently a significant contributor to the association between bimanual hand use and peduncle asymmetry. For unimanual capacity, age was a significant contributor to this relationship [model *F*(6,28) = 12.35, *p* < 0.0001, *r* = 0.85, *R* ^2^ = 0.73, *t*(age) = 2.7, *p*(age) = 0.017]. The time to complete the JTTHF was lower in older children than younger children, indicating better unimanual capacity.

The interaction between age and peduncle asymmetry is also highly correlated with baseline JTTHF [*F*(1,33) = 20.51, *p* < 0.0001, *r* = 0.62, *R* ^2^ = 0.38] as well as baseline AHA [*F*(1,33) = 8.41, *p* = 0.007, *r* = 0.45, *R* ^2^ = 0.20]. This means that the relationship between peduncle asymmetry and baseline hand function differs with age.

### Peduncle asymmetry does not predict improvement in hand function

**Fig. 3:**
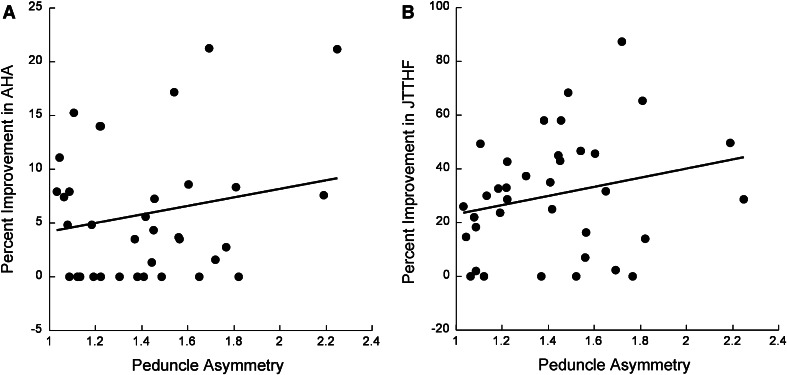
Response to therapy is not linearly associated with peduncle asymmetry. **a** Percent improvement in AHA was not significantly linearly correlated with peduncle asymmetry [*F*(1,33) = 1.29, *p* > 0.2, *R* ^2^ = 0.038]. **b** Percent improvement in JTTHF was not significantly linearly correlated with peduncle asymmetry [*F*(1,33) = 2.72, *p* > 0.11, *R* ^2^ = 0.074]

We examined the linear association between the percent improvement in AHA and JTTHF and peduncle asymmetry. The linear association between peduncle asymmetry and percent improvement in AHA was not significant [Fig. 3a, *F*(1,33) = 1.29, *p* = 0.26, *R* ^2^ = 0.038]. Similarly, there was not a significant linear association between peduncle asymmetry and percent improvement in JTTHF [Fig. 3b, *F*(1,33) = 2.72, *p* = 0.11, *R* ^2^ = 0.074].

To further examine any possible relationships between peduncle asymmetry and improvement in hand function, we used multivariable statistics to determine the effects of the following covariates: initial severity, age, gender, side of lesion, lesion type, and the lag time from when the MRI was done to the time of training. The inclusion of covariates did not significantly strengthen the association between percent improvement in AHA and peduncle asymmetry [*F*(7,27) = 1.16, *p* = 0.36, *r* = 0.48, adjusted *R* ^2^ = 0.032, *t* < 1.0, *p* > 0.1 for all covariates]. Likewise, including these covariates in a regression model did not reveal a significant association between percent improvement in JTTHF and peduncle asymmetry [*F*(7,27) = 0.86, *p* = 0.55, *r* = 0.43, adjusted *R* ^2^ = 0.03, *t* < 1.4, *p* > 0.1 for all covariates].

We also normalized the peduncle size relative to the whole brain cross-sectional area and tested the association between normalized peduncle asymmetry and hand function improvements. There were no significant associations between normalized peduncle asymmetry and AHA improvement [*F*(1,33) = 0.15, *p* = 0.90] or JTTHF improvement [*F*(1,33) = 1.69, *p* = 0.20].

To further test the robustness of this result, we compared the relationship between recovery and peduncle asymmetry in children who were within the top 75 % (“strong responders”) in terms of improvement in AHA (*n* = 23) or JTTHF (*n* = 24) after training, or those in the lower 25 % of recovery (“weak responders”). The association between peduncle asymmetry and percent improvement was not statistically significant in the strong responders [AHA: *F*(1,21) = 0.23, *p* = 0.63, JTTHF: *F*(1,22) = 2.96, *p* = 0.10] or the weak responders [AHA: *F*(1,9) = 2.5, *p* = 0.15, JTTHF: *F*(1,8) = 0.13, *p* = 0.9]. Thus, response to intensive hand upper extremity training is not linearly associated with peduncle asymmetry.

## Discussion

We examined the association between an estimate of CST dysgenesis, peduncle asymmetry, and unimanual and bimanual skill in children with congenital unilateral CP. Since previous studies described a linear association between CST damage and impairment in manual skill, in children with CP and adults with stroke (Bleyenheuft et al. 2007; Bouza et al. 1994; Duque et al. 2003; Feys et al. 2010; Mark et al. 2008), we hypothesized that peduncle asymmetry would be associated with baseline hand function and the capacity for recovery with intensive training.

Consistent with prior studies, we found that baseline unimanual capacity, measured by the JTTHF, was strongly linearly associated with peduncle asymmetry (Bleyenheuft et al. 2007; Duque et al. 2003). The magnitude and strength of the association found in this study were similar to previous studies. We also showed that bimanual hand use, measured by the AHA, is associated with peduncle asymmetry. Even though prior studies used different measures of hand function than the JTTHF and AHA, the linear association between CST dysgenesis and manual skill deficits was a consistent finding. It is important to note that neither the JTTHF nor the AHA measure how the task is performed (e.g., kinematics).

Contrary to our hypothesis, we found that there was no linear association between peduncle asymmetry and the capacity of children to improve unimanual or bimanual hand function with HABIT. The independence of functional improvement and peduncle asymmetry held despite correction of a large number of covariates, including lesion type, age at time of HABIT, age at MRI, and brain size. This represents an exciting, novel finding, indicating that children with a range of severity in peduncle asymmetry can benefit from HABIT.

Two studies have examined the relationship between MRI measures and improvement in hand abilities after CIMT in children with unilateral CP and adults with chronic stroke. Rocca et al. (2013) found a significant association between integrity of fibers in the lesion area and improvement in hand function after CIMT. In contrast, Rickards et al. (2013) did not find a significant association between improvement after CIMT and CST integrity in children or adults. Both studies enrolled a low number of participants (*n* < 30). Further research regarding the predictive power of MRI features and recovery after intensive hand training is needed.

Our results suggest that improvements in hand function after training are not exclusively mediated by the contralaterally projecting CST. Other motor pathways, including ipsilateral connections from the less-affected M1, are possibly important in recovery. Developmental studies have suggested that lesions occurring earlier in life are more likely to result in increased ipsilateral connections from the less-affected M1 (Fowler et al. 2010; Kuhnke et al. 2008; Tian et al. 2010; Holmstrom et al. 2010; Yoshida et al. 2010; Eyre 2003), likely by preserving the bilateral CST projections that occur during development (Staudt 2010). Lesions resulting in strong ipsilateral connections also can produce more severe deficits in hand function, inviting speculation that these ipsilateral connections are maladaptive (Kuhnke et al. 2008). Laboratory models of injury have also shown that other pathways can be involved in motor recovery (Z’Graggen et al. 1998; Carmel et al. 2013). Moreover, Rose et al. (2011) examined the relationship between hand function and asymmetry of corticospinal and corticothalamic pathways in children with unilateral CP. They did not find a significant association between CST asymmetry and hand function, but instead found that asymmetry of the corticothalamic pathway was strongly correlated with hand function. Thus, it is possible that integrity of corticothalamic fibers may be related to improvement after intensive hand therapy.

Lesion type was not a significant predictor of baseline hand function or amount of recovery. Some studies indicate a relationship between lesion type and hand function, suggesting that children with periventricular lesions have better hand function than children with cortical or subcortical lesions (Cioni et al. 1999; Feys et al. 2010). It is possible that we failed to find an effect of lesion type on hand function since children in this study had a variety of lesion types. Therefore, we potentially lacked the power to discern differences based on lesion type.

A limitation of our study is that structural images were used. Some studies have used structural images to examine motor pathway dysgenesis in children with CP (Duque et al. 2003) and adult stroke patients (Pineiro et al. 2000). Our regression model of baseline hand function shows highly similar results as the Duque et al. study (2003), suggesting that these findings are reproducible. An important study compared peduncle asymmetry analysis from structural scans to analysis using DTI to identify the CST (Bleyenheuft et al. 2007). In this study, DTI was more effective at finding associations between motor symptoms and neuroanatomy than peduncle asymmetry on structural scans. When the peduncle size is measured, the size includes not only the CST but also other tracts and the substantia nigra. Thus, peduncle measure is an estimate of CST dysgenesis but is not as precise as a DTI-based analysis. Bleyenheuft et al. suggested that peduncle measures systematically underestimate CST dysgenesis.

Moreover, structural images provide no information about the integrity of neural pathways. Indeed, the integrity of motor pathways may be more predictive of the potential for recovery than the estimated size of motor pathways. DTI provides information about the integrity of fibers by determining the anisotropy of fibers, a measure of directional diffusivity of water along a fiber bundle. Anisotropy of motor pathways is lower than normal in children with CP (Bleyenheuft et al. 2007; Thomas et al. 2005; Yoshida et al. 2010). Future studies should examine the relationship between DTI-determined motor fiber integrity and potential for improvement in hand function.

Another limitation of our study is that for many children, there was a lag time between the time at which the MRI was taken and the time of intervention (average 42.5 months). Although lag time between MRI and training was not a significant variable in the regression models, it is possible that children’s peduncle asymmetry had changed between the time of MRI acquisition and training. Two pieces of evidence suggest this is not the case. First, there was a strong relationship between peduncle asymmetry at the time of MRI and baseline hand function. Second, the asymmetry of the peduncles remained remarkably stable over time in the children who had repeated MRI scans. Further study of CST development, particularly in early development, will help to explore how CST anatomy changes in early development, as has been done with physiological measures of CST function (Eyre et al. 2007; Staudt 2010). Additionally, we only had sufficient power to test linear relationships between variables. It is possible that a more complex nonlinear relationship might exist between peduncle asymmetry and recovery.

This study is limited to children with unilateral CP who have mild to moderate impairments of the affected hand. Our finding that the independence of recovery from peduncle asymmetry invites the possibility that children with a great amount of peduncle dysgenesis, and more severe hand deficits than children studied here, might respond to intensive treatment. It is likely that other factors are stronger predictors of treatment efficacy, such as intensity of training, treatment schedule, and attentiveness of the child during therapy. Moreover, other neurological factors that were not assessed in this study could be predictors of recovery. For example, neurological damage that impairs vision, attention, or cognitive potential may impact recovery, as children with these impairments may be limited in their ability to participate in intensive training protocols. Future studies that can identify predictors of recovery will be extremely valuable, so that treatment can be tailored to children based on these parameters.

## Abbreviations

AHA: Assisting hand assessment
CM: Cortical malformation
CP: Cerebral palsy
C/SC: Cortical/subcortical lesion
CST: Corticospinal tract
DTI: Diffusion tensor imaging
HABIT: Hand–arm bimanual intensive therapy
JTTHF: Jebsen–Taylor test of hand function
M1: Primary motor cortex
MRI: Magnetic resonance imaging
PV: Periventricular injury
